# Supplementary material for: Synapse types are spatially associated with regional hemodynamics in the mouse brain
Source: PLoS Biol. 2026 Feb 12;24(2):e3003637. doi: 10.1371/journal.pbio.3003637 (PMC12919930; doi:10.1371/journal.pbio.3003637)
Supplement: Attachment — Submitted filename: Hansen_Response_to_Reviewers.pdf [file pbio.3003637.s022.pdf]

Dear Dr. Hart and Reviewers,

Thank you for the constructive feedback on our first submission, and for the opportunity to revise the manuscript. Following your comments and suggestions, we have thoroughly revised the manuscript. In this letter, we respond to each of the reviewers' comments in detail. Reviewer comments are in **bold font** and our responses are in regular font.

Major changes include: (1) we clarify the intended scope of our work as a research rather than resource article (highlighted by Reviewer #3); (2) we remove any instances of causal language and clarify the correlational nature of our findings (highlighted by Reviewer #1); and (3) we demonstrate the sensitivity and robustness of the analyses by testing effects of global signal regression as well as reproducibility of time-series features across space and individuals (highlighted by all three Reviewers).

Reviewer #1:

In this article, the authors aim to make an important link between microscale synaptic events and their functional embedding in global changes of brain dynamics, by leveraging advanced and cutting-edge techniques and conducting a thorough investigation and validation across multiple fMRI datasets. The main findings revolve around how specific synapse types are associated with specific features in (f)MRI dynamics and structure. This article is well written and clear in its logic and findings. I particularly appreciate the direct questions guiding each section and reminding the reader of the main goal of each subpart. I also appreciate seeking validation in other datasets, and across brain states. A few comments may help frame the results.

1. Overall, I am not fully familiar with the hctsa tool for timeseries features extraction. Yet, I feel the article often conflates correlation with causation. For example, stating that certain synapse types "shape" neural dynamics:

a. suggests a causal relationship, which cannot be firmly concluded from correlative analyses. Even more so since—as far as I understand it—this data is correlating with task-free resting state data, so it's looking at spontaneous, not evoked, fluctuations.

b. defines 'BOLD-fMRI' fluctuations as 'neural signal' which isn't entirely biologically true. The BOLD signal of fMRI is not a direct measure of underlying neuronal changes, and even when taking into account a hemodynamic response, this may change its shape across different brain regions/brain states.

The language should be more cautious and recognize the correlative nature of the findings, or if there is a de facto causal relationship, this should be stated more clearly for audience not familiar with the hctsa tool and the features extracted.

We fully agree with the Reviewer and have thoroughly revised the text to remove causal language. In particular we avoid referring to fMRI activity as "neural", and we have replaced instances of "shape" with more accurate language (e.g. "relates to").

Title:

> "Synaptome architecture shapes regional dynamics in the mouse brain" → "Spatial associations between synaptic architecture and regional dynamics in the mouse brain"

Abstract:

> “How do diverse synapses ~~shape~~ relate to the spatial patterning of whole-brain dynamics?”

> “We find that specific synapse types ~~shape~~ are associated with specific features of haemodynamics ~~dynamics~~, including high-amplitude events and signal stationarity.”

> “Collectively, this work ~~demonstrates~~ suggests that the spatial organization of microscale synapse types ~~fundamentally~~ may shape whole-brain dynamics.”

Introduction:

> “Throughout, we find that the differential expression of synapse types maps onto unique features of macroscale ~~neural~~ haemodynamics and interregional connectivity.”

Results:

> “To address this question, we analyze spontaneous fMRI activity in awake mice (N=10), and compare regional variation in ~~neural~~ haemodynamics to regional variation in synapse types.”

> “Lastly, while we do repeat these analyses for short-lifetime PSD95 synapses (Fig. S7), we find a poor alignment between synapse density and time-series features, suggesting that short-lifetime PSD95 synapses are not reliably associated with specific features of ~~neural~~ haemodynamics.”

> “Thus far, short-lifetime PSD95 synapses consistently demonstrate weak or no correlation with features of macroscale ~~neural~~ haemodynamics, anatomical connectivity, and functional connectivity (Fig. S7). ”

Discussion:

> “As a result, the synaptic composition of a brain region is not only ~~shapes~~ associated with its macroscale dynamics but also its embedding in whole-brain structural and functional networks.”

2. Figure 1, the overlay of synaptic density plots onto the brain are performed for the right hemisphere only, and mirrored to the left. It would be useful to know the reason for this choice: is it commonly assumed that synaptic density is specular in both hemispheres, even knowing that structurally and functionally the brain is asymmetric? (there are multiple references in rodent fMRI literature for this).

Apologies for the unclear figure caption. The synapse density data is hemisphere-specific, and we analyze both hemispheres whenever possible. However, the Allen Mouse Atlas tract-tracing structural connectivity data was only collected in the right hemisphere. For this reason, when we show synapse density data in the structural parcellation (137 right hemisphere regions; left panel of Figure 1 e, f, g), we mirror the right hemisphere synapse density data onto the left hemisphere to better visualize brain structures. Conversely, synapse density data shown in the functional parcellation (88 bilateral regions; right panel of Figure 1 e, f, g) is bilateral and therefore differs across hemispheres.

That said, in this dataset, synapse density is consistently highly symmetric for long-lifetime PSD95 (mean r = 0.92), SAP102 (mean r = 0.89), and colocalized synapses (mean r = 0.90). Short-lifetime PSD95 synapses demonstrate slightly lower inter-hemispheric symmetry (mean r = 0.71).

Figure S19:

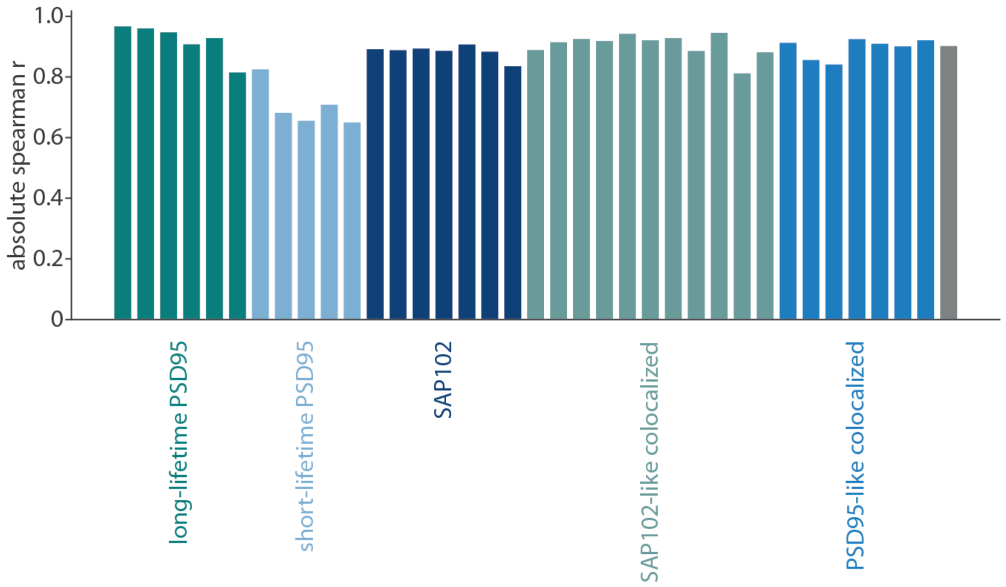

> "Fig S19. Synapse density is symmetric across hemispheres | We correlate right and left hemisphere synapse density for all 37 synapse subtypes."

We have edited the caption of Figure 1 to clarify this point.

Figure 1 caption:

> "Note that, since tract-tracing data is limited to the right hemisphere, the structural parcellation encompasses 137 right hemisphere regions. We show mirrored data on the left hemisphere."

3. Figure 2d, I personally struggle to understand what this graph is trying to show.

We agree with the Reviewer that the original caption describing this graph is unnecessarily vague. We have expanded the caption as follows:

Figure 2d:

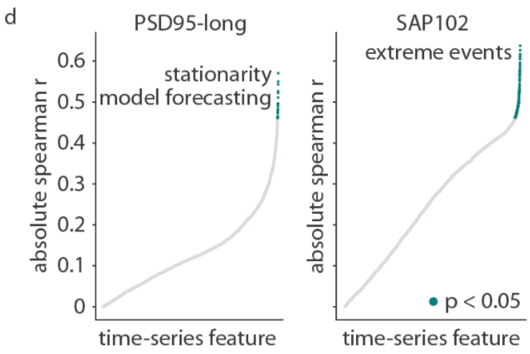

“(d) Each time-series feature was correlated (Spearman's r) with synapse density, for long-lifetime PSD95 and SAP102 synapses separately. Each point on the scatter plots represent a correlation coefficient, where green points indicate statistical significance after multiple comparisons correction (p<0.05, Bonferroni-corrected). Time-series features were sorted by correlation magnitude, and a selection of key, statistically-significant features are listed in the figure.”

4. I really appreciate the transparency and clarity of the authors in terms of limitations in their work, one of these being the focus on two synaptic types. Yet, to generalize from two proteins to the entire "synaptome" and its influence on causally shaping whole-brain dynamics is an overstatement.

We agree and have toned down the language in the text (also in response to the Reviewer’s Comment #1). In the revised manuscript, the term “synaptome” is now only used to introduce the concept of synaptic diversity, and to discuss future research avenues for understanding the brain’s synaptome.

5. Along the lines of the limitations of this work, the authors clearly state the major issue being the synaptic density analysis performed on N=1 male mouse. First of all, I appreciate keeping a consistent analysis with only utilizing male mice for the fMRI datasets. While I do understand that this article is an initial stepping-stone to create a bridge between micro-scale and macro-functional reorganization (i.e., reproducing all these analyses on a second, third, fourth mouse falls outside the scope of this specific article), it would be informative to investigate how these results relate to the fMRI profiles of female mice.

We agree with the Reviewer that understanding how the findings generalize to female mice would be informative. Due to its recent methodological development, awake resting-state fMRI data have not yet been recorded in female mice. Furthermore, we are not aware of any systematic comparisons of awake fMRI dynamics in male and female mice. The lack of awake fMRI data in female mice is not only a limitation in our work but a broader gap in the study of brain activity in animal models. We hope that the availability of our code and data on Github will make it possible to seamlessly rerun the analyses using data from female mice, once it is collected and available.

We now discuss this limitation in the text (“Discussion” section, Paragraph #10):

> “Second, although whole-brain synapse mapping technology is both recent and state-of-the-art, current whole-brain synapse density maps are limited to a single male mouse. This is an important limitation that hinders our ability to assess whether the current findings can be generalized to a broader population of animals or across sexes.

> As synapse mapping technology becomes more high throughput, it will become possible to study how synaptic architecture influences individual-specific structure, function, and behaviour. Similarly, time-series feature calculation was conducted on fMRI data from a small sample of 10 mice, and individual reproducibility of the spatial distribution of time-series features is poor (mean r=0.13). We therefore average time-series features across mice to maximize signal and reproducibility (predicted mean r=0.42; see Methods for details). In addition, fMRI data were acquired in exclusively male mice, precluding an understanding of how these findings generalize to female mice.”

6. Similarly to #5, commenting on the age differences in the PND80 mouse and the fMRI datasets (were they all the same age?) would help contextualize the results. Sorry if I missed this.

We thank the Reviewer for bringing up this point as we neglected to include age information in the original manuscript’s Methods section! As mentioned by the Reviewer, synapse density data was acquired in a PND80 mouse. The fMRI data were acquired in adult mice (all <6 months) of varying ages. Sample mean and standard deviations are: 141.4 +/- 21.2 days (awake state), 77.4 +/- 6.9 days (halothane anaesthetized state), 168 +/- 57.4 days (medetomidine-isofluorane anaesthetized state).

Therefore, the halothane anaesthetized mice are closest in age to the PND80 mouse. However, given the consistent findings across halothane (~PND77) and medetomidine-isofluorane (~PND168) anaesthetized mice, we do not expect our findings to be significantly impacted by age.

We have added the sample ages to the Methods (“Methods” section, “fMRI data acquisition” subsection, Paragraph #1):

> “Resting-state fMRI scans were acquired by Gutierrez-Barragn et al 2022 in 10 awake, head-fixed C57BL/6J male mice (mean +/- standard deviation of age = 141.4 +/- 21.2 days; full methodological details available in Gutierrez-Barragan et al 2022).”

Paragraph #3:

> “Two additional groups of age matched male C57BL/6J mice were scanned under anaesthesia in Gutierrez-Barragan et al 2022. The first group includes 19 mice (mean +/- standard deviation age = 77.4 +/- 6.9 days) scanned under shallow halothane anesthesia (0.75%) (Gutierrez-Barragan et al 2019). The second group includes 14 mice (mean +/- standard deviation age = 168 +/- 57.4 days) scanned under medetomidine-isofluorane anesthesia (0.05mg/kg bolus and 0.1mg/kg/h IV infusion, plus 0.5% isofluorane).”

7. These are more technical questions, out of curiosity (therefore optional). The slice thickness of the fMRI data shows quite thick slices (600 um in one direction). Can this bias the results, compared to having thinner slices? How robust are the results with and without gsr? I am also very curious in single-mouse fMRI results: how variable are those from the average utilized?

We thank the Reviewer for these technical questions as it prompted valuable robustness analyses that have now been added to the manuscript.

Re slice thickness:
The choice of 600 μm slice thickness reflects a deliberate trade-off, as our focus on dynamic mapping required prioritizing temporal rather than spatial resolution. Importantly, prior work has consistently shown

that resting-state fMRI networks in the mouse brain are spatially distributed across multiple antero-posterior cortical and subcortical regions (e.g., Gutierrez-Barragan et al., Current Biology, 2019; 2022; Grandjean et al., 2020). These observations suggest that the mesoscale spatial resolution achieved here is sufficient to capture the distributed topography (and corresponding dynamic organization) of these networks. In this light, the selected protocol reflects a pragmatic optimization of spatiotemporal resolution that is well established in rodent fMRI, and we do not expect slice thickness to bias any of our findings.

Re Global Signal Regression (GSR):
We have now removed the global signal from all time-series, re-ran the time-series phenotyping pipeline, and correlated the spatial distributions of time-series features with synapse density brain maps. First, we find a close correspondence between time-series feature value before and after GSR (Spearman r = 0.75, p~0). Below is a new Supplementary Figure S16 showing the correlation between every brain region's time-series feature value before and after GSR (6411 time-series features x 88 brain regions = 564,168 observations). Second, when we re-correlate each time-series feature with synapse density, we find consistent correlations across all synapse types. The time-series features that are least consistently correlated with synapse density are those where -0.2 < r < 0.2. Meanwhile, all highly correlated time-series features remain highly correlated regardless of GSR.

Re single-mouse versus group-average time-series feature profiles:
Finally, we compare the group-average time-series phenotypes used throughout the analyses with single-mouse time-series phenotypes (Figure S17). More specifically, for every brain region (rows) and mouse (columns), we correlate the full regional time-series profile of the mouse with the average regional time-series profile. We show the correlation coefficients in the heatmaps below. All individual time-series phenotypes are positively correlated with the mean time-series phenotype, with the most variable brain region being correlated on average at r=0.33 and the least variable brain region being correlated on average at r=0.68.

These findings are encouraging and point to the robustness of both awake rodent fMRI and time-series phenotypes. We nonetheless address the limitations of time-series feature variability and small sample size in the manuscript (see also our response to Reviewer #3 Comment #3):

("Results" section, "Sensitivity and robustness analysis" subsection, Paragraph #1):

> "In this final subsection, we conduct six analyses to gauge the sensitivity and robustness of the current findings: (1) we confirm synapse density data is coexpressed with synapse-related gene expression data from a larger population of mice, (2) we confirm our findings are not driven by fMRI SNR, (3) we ensure fMRI signal amplitude and time-series features are not driven by motion, (4) we confirm our findings remain consistent when global signal regression is applied to the fMRI signal, (5) we assess the replicability of regional time-series phenotypes and spatial time-series feature value distributions, and (6) we confirm that these results are specific to synapse types as opposed to spatial variation in cell types."

(Paragraph #6):

> "Fourth, we ensure the relationship between synapse density and dynamics is invariant to global signal regression. We apply global signal regression (GSR) to the fMRI time-series of each mouse and recompute the complete time-series phenotype (i.e. Fig. 2c). We find that the time-series phenotype from global signal regressed and non-global signal

regressed time-series are highly correlated (r=0.75, p~0; Fig. S16a). Next, we re-correlate each time-series feature with all five synapse types. Overall, we find that synapse-related time-series profiles are highly consistent (0.58 < r < 0.90, Fig. S16b). These analyses demonstrate that time-series phenotypes are robust to GSR.

Fifth, we assess the reproducibility of time-series features by comparing individual-mouse phenotypes to group-average phenotypes. For every brain region and mouse, we correlate the full regional time-series phenotype profile of the mouse with the average regional time-series phenotype profile (6,471 x 1 vector of time-series feature values) (Figure S17). All individual time-series phenotypes are positively correlated with the mean time-series phenotype (range of mean correlation: [0.33, 0.68]). Next, for every time-series feature and mouse, we correlate the spatial distribution of time-series feature with the average time-series feature. The empirical mean correlation between individual and group time-series spatial distributions is 0.43 (see Methods for more details)."

("Discussion" section, Paragraph #10):

"Similarly, time-series feature calculation was conducted on fMRI data from a small sample of 10 mice, and individual reproducibility of the spatial distribution of time-series features is poor (mean r=0.13). We therefore average time-series features across mice to maximize signal and reproducibility (predicted mean r=0.42; see *Methods* for details)."

Figure S16:

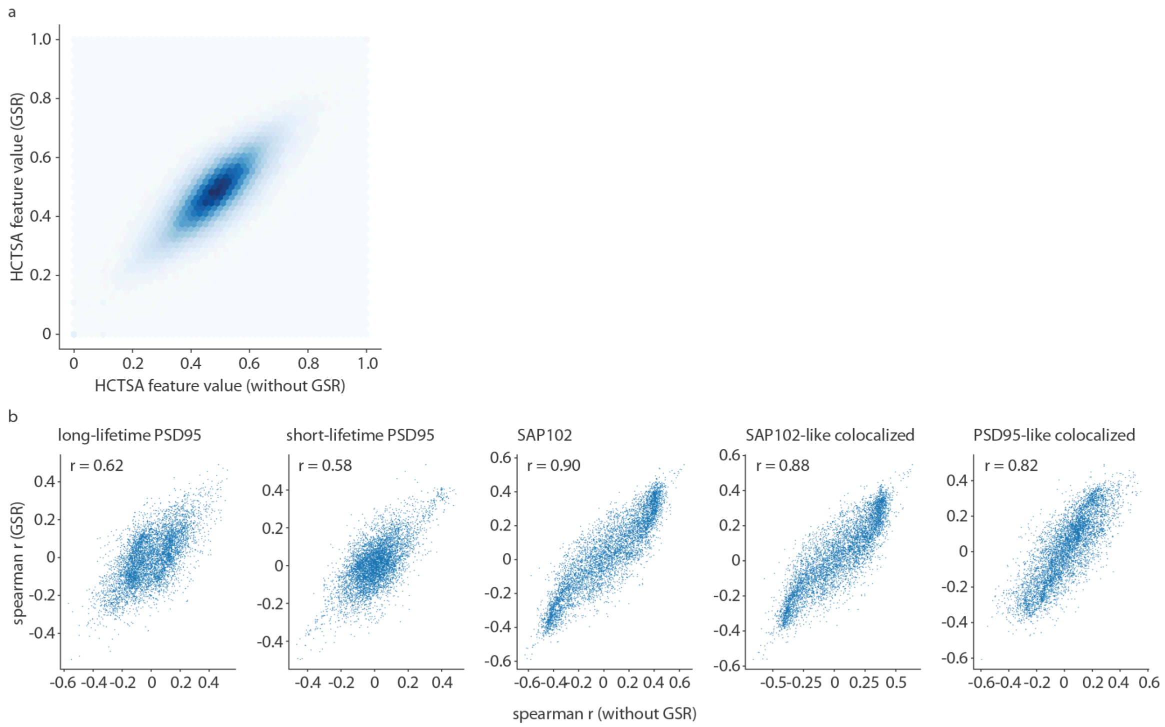

"Figure S16. Temporal features are robust to global signal regression (GSR) | The analysis in Figure 2 was repeated after applying GSR to all regional time-series. (a) Time-series feature values without (x-axis) and with (y-axis) GSR are highly correlated

(r=0.75). (b) Each synapse type is associated with a time-series phenotype (defined as the Spearman correlation between spatial distributions of synapse type and time-series features; Figure 2d). We correlate time-series phenotypes before and after GSR.”

Figure S17:

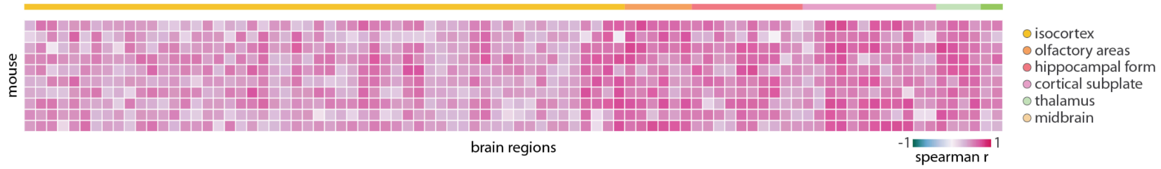

“Figure S17. Each brain region is associated with a time-series phenotype (6,471 x 1 vector of time-series values). This heatmap represents the Spearman correlation between each region’s group-averaged time-series phenotype with the time-series phenotypes from individual mice. Brain regions are ordered according to ontological structure, as indicated by the vertical bar and the legend.”

8. This is personal preference, but I find the color scale chosen to be very hard to interpret and follow, especially in the figures where both ROIs and density are shown, which basically have overlapping colors. Unless this is made on purpose (for which, please make it clearer), it would perhaps be more visually striking to use a different color scheme to categorize ROIs, rather than using a similar shades of blue/greens of the intensity. I understand it can be troublesome to edit all figures at this stage, so this is optional.

We thank the Reviewer for bringing this concern to our attention, which was also flagged by Reviewer #2 (see a similar response to Reviewer #2 Comment #2). We have opted to change the categorical colour scheme for regional ontology while the blue-green continuous colourmap remains the same. We hope the warm-toned categorical colourmap makes it much easier to distinguish between synaptic densities and regional ontologies. As a result of this change, all main and supplementary figures have been updated.

Figure 2 as an example:

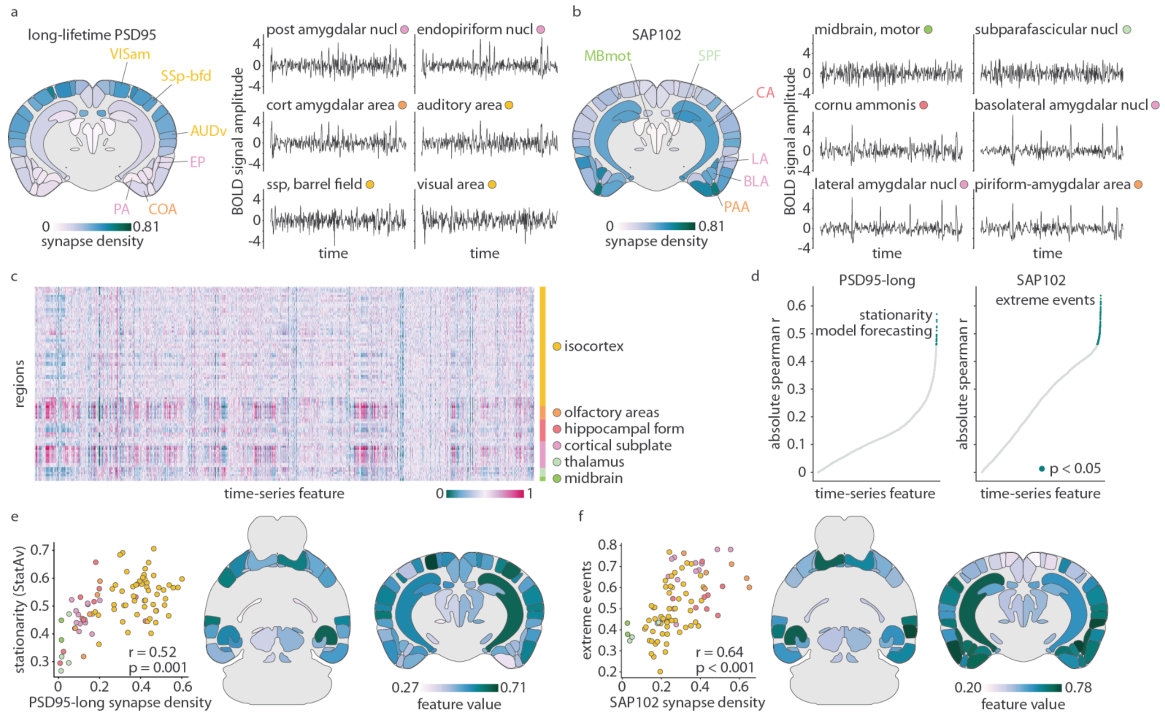

Minor:

9. Fig. 2. BOLD signal amplitude doesn't have a y axis range. Are those timeseries all varying within the same range?

Yes the y-axes are all identical, and each time-series is z-scored. We have added in the z-scores on the y-axis in Figure 2. We now mention this in the figure caption.

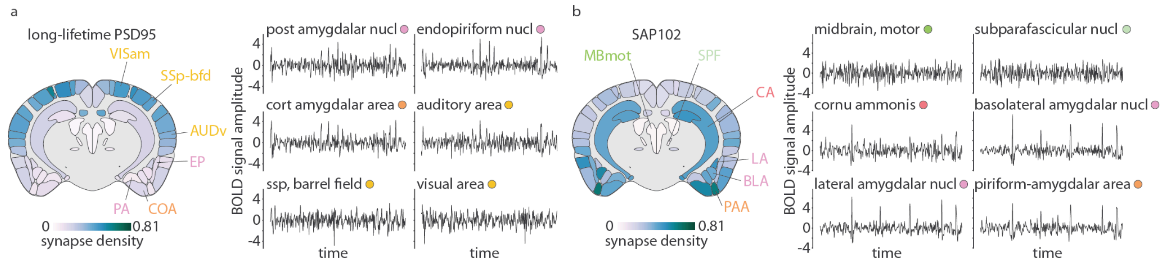

> "(a--b) Six example z-scored time-series from brain regions with variable long-lifetime PSD95 (a) and SAP102 (b) synapse density."

10. Throughout the article, results are reported across the anatomically defined brain regions, one of which is the isocortex. However, 'isocortex' includes a lot of highly specialized and diverse types of regions, which I assume may show different behavior in this work. A deeper dive into what each subregion (Sensory compared to Motor for example) does, would be interesting, especially in the comparison between awake and anesthesia, where I assume differences within and across these regions were found in the fMRI functional connectivity analyses.

This is an interesting question we had not previously considered. We have now rerun the analysis comparing structure-function coupling in awake and anaesthetized mice across all three synapse types. We stratify isocortical regions according to two methods: (1) we apply structural and functional connectivity networks defined by Coletta et al 2020 Science Advances, and (2) we manually generate functional groups by organizing isocortical regions according to the functional system mentioned in the anatomical name (e.g. “Primary motor area” is labelled “motor”, “primary somatosensory area, barrel field” is labelled “somatosensory”). We note however that, while we show these results in the supplement, we refrain from overinterpreting as the number of observations within each network is small (max n = 12) and therefore formal statistical analyses were not performed.

Our original finding was that short lifetime PSD95 synapses only improve SC-FC coupling when mice are awake. We interpret this to mean that short lifetime PSD95 synapses are recruited most during the awake state. By stratifying our findings within cortical networks, we are able to more specifically pinpoint the systems that demonstrate this distinction. We find that, in awake mice, short-lifetime PSD95 synapses especially improve SC-FC coupling in visual, cingulate, limbic, and retrosplenial networks (broadly aligned with the default mode network in Coletta et al 2020). Meanwhile, in motor, somatosensory, and insular networks, (broadly aligned with “latero-cortical network” in Coletta et al 2020), there is little difference in ΔR^2_{adj} across awake and anaesthetized states. Furthermore, while SAP102 synapses show consistent ΔR^2_{adj} across awake and anaesthetized states regardless of functional network, long-lifetime PSD95 synapses show *increased* ΔR^2_{adj} in auditory and visual networks.

To summarize, SAP102 synapses appear to be recruited in the same manner regardless of whether or not the mouse is awake; long-lifetime PSD95 synapses improve SC-FC coupling most in the somatosensory network (regardless of awake or anaesthetized state), but also improve SC-FC coupling more in the anaesthetized state within auditory and visual networks; and lastly short-lifetime PSD95 synapses demonstrate broadly decreased ΔR^2_{adj} in the anaesthetized state, although not in motor, somatosensory, nor insular networks.

We have included a new Supplementary Figure S9:

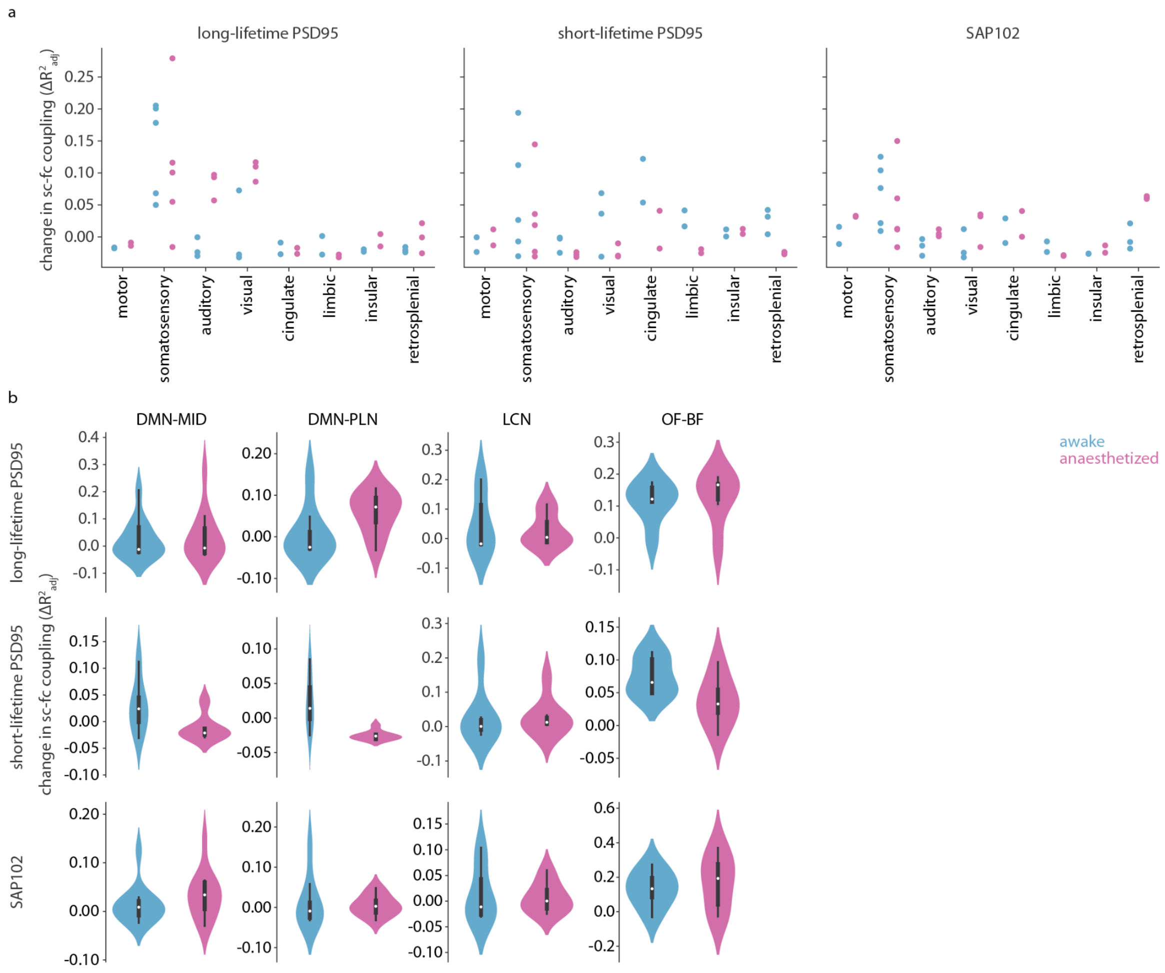

“Figure S9. Awake vs anaesthetized change in structure-function coupling (ΔR^2_{adj}) stratified by functional systems | We compare the change in structure-function coupling (distribution of ΔR^2_{adj}) in awake versus anaesthetized mice, for all three synapse types (originally shown in Figure 4c), stratified by two network systems. (a) ΔR^2_{adj} is stratified by functional system included in region name (e.g. “Primary motor area” is labelled “motor”, “Primary somatosensory area, barrel field” is labelled “somatosensory”). (b) ΔR^2_{adj} is stratified by structural and functional connectivity networks defined by Coletta et al 2020 Science Advances. DMN-MID = midline DMN; DMN-PLN = posterior-lateral DMN; LCN = latero-cortical network.”

11. The integration of datasets from different sources and spatial resolutions necessitates downsampling the high-resolution synapse data. This is a necessary step, but it means that much of the fine-grained spatial detail from the original synaptome maps is partially lost. A brief mention of this as a methodological limitation in the discussion would be appropriate.

Agreed:

(“Discussion” section, Paragraph #10):

> “Third, synapse density, tract-tracing and fMRI data were derived in separate mouse populations, highlighting the need for more comprehensive datasets that include measurements from diverse scales of brain organization. Furthermore, integrating measurements across spatial scales necessitated downsampling the high resolution synapse density data.”

12. I believe anesthezia should be spelled with an s.

Fixed.

Reviewer #2:

This work builds on previous studies by the authors regarding the characterization of excitatory synapses, in which they used the expression of proteins (PSD95 and SAP102) and the morphology of individual synapses for the identification of synapse types or "synaptomes". The present work analyzes publicly available datasets on synapse density (Zhu et al., 2018) and synapse protein lifetime (Bulovaite et al. 2022) of the identified synapse types, as well as mouse fMRI data (Gutierrez-Barragan et al. 2022) and structural and gene expression data from the Allen Mouse Brain Connectivity Atlas and the Allen Mouse Brain Atlas respectively (and their related publications). Using this approach, this study focuses on the regional heterogeneity of the synapse types and correlates it with features of region-specific fMRI dynamics, and region-specific anatomical and functional connectivity. It further identifies differential roles of synapse types in shaping the structure-function coupling across the different animal states. Overall, this work connects the microscale (synaptic profiles) to the macroscale (inter regional dynamics) and provides several insights into how different synapses shape brain function. Importantly, this work is a very nice example of how open science allows the integration of work done across labs/institutes. Following are my comments on the manuscript, in order of appearance in the text.

Comments
1. Fig. 1a and Fig. S2a are the same plot (and the same as Fig. 3d of Zhu et al.). Likewise, Fig. S2b is the same as Fig. 1b (with some missing labels, e.g., although the y-axis label of S2b pools together the colocalized synapse subtype distributions, the delineated boxes correspond to the PSD95- and SAP102-like cases). Consider removing redundant plotting.

We have removed Figure S1a and b (previously Figure S2 a and b).

2. Figure 1 and elsewhere: The chosen color pallets difficult to follow. The colors used to indicate values (e.g., density or correlation in Fig. 1) are very similar to the colors used to indicated the region's ontological structures (Fig 1c-g). This is especially confusing in Fig. 2a,b where values (synapse density) are shown in coronal slices (using a different color pallet from Fig. 1a for synapse density), while the region ontological structure on the right panels of these subplots use very similar colors. In the same figure, time-series features are shown using a different color scheme in panels 1c and 1e/f - slices. For the scatter plots of Fig 2e,f, do the colors indicate regions? Overall, consider adjusting the color pallets for consistency and discriminability.

We thank the Reviewer for bringing this concern to our attention, which was also flagged by Reviewer #1 (see a similar response to Reviewer #1 Comment #8). We have opted to change the categorical colour scheme for regional ontology while the blue-green continuous colourmap remains the same. We hope the warm-toned categorical colourmap makes it much easier to distinguish between synaptic densities and regional ontologies. As a result of this change, all main and supplementary figures have been updated.

Figure 2 as an example:

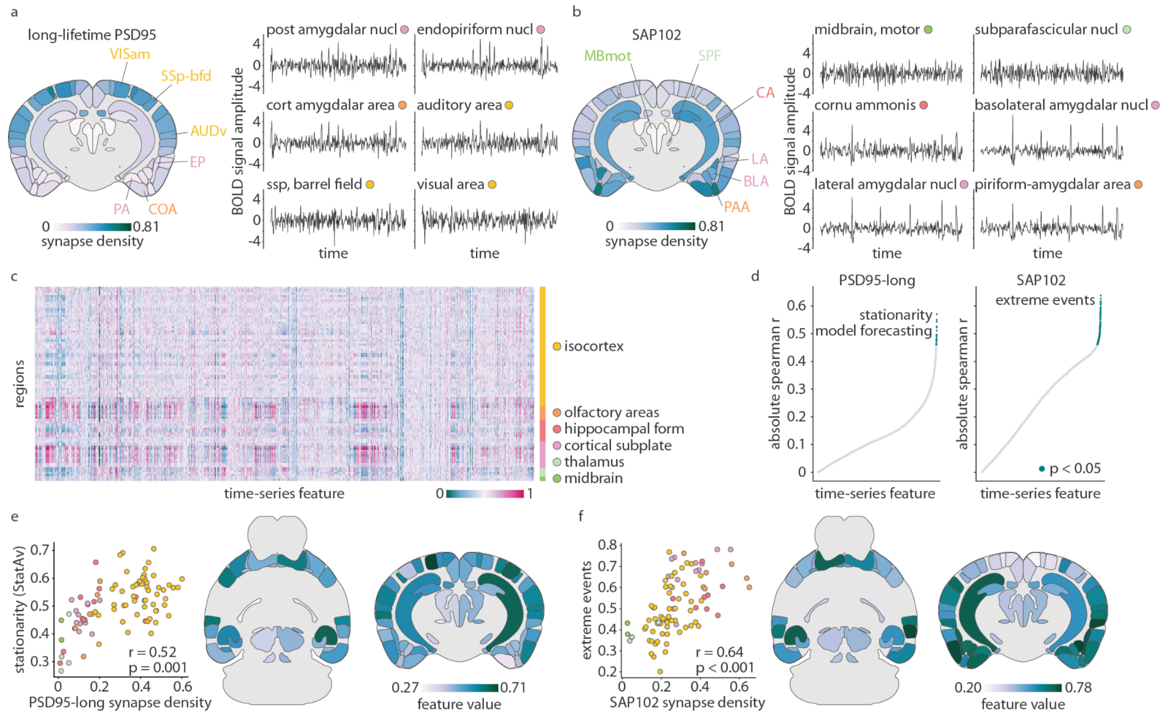

3. Figures S3-S6: Information about the delineated boxes is missing from the figure legends. In the methods section, how was the number of clusters determined when using the agglomerative clustering method (i.e., what was the stopping criterion)?

We thank the Reviewer for bringing this to our attention. When using the agglomerative clustering method, we did not use a stopping criterion; rather, the number of clusters was defined a priori. We ran the algorithm across 2 through 10 clusters. Clustering solutions were visually inspected, and the chosen clustering solution was selected based on (1) reducing the number of clusters with very few (<5) features, (2) maximizing correlations within clusters, (3) minimizing correlations between clusters. That said, the interpretation of clustering solutions are all very similar since highly correlated features remain within one cluster while poorly correlated features are broken into progressively smaller clusters (e.g. see figure below which contains all 9 clustering solutions for long-lifetime PSD95 synapses).

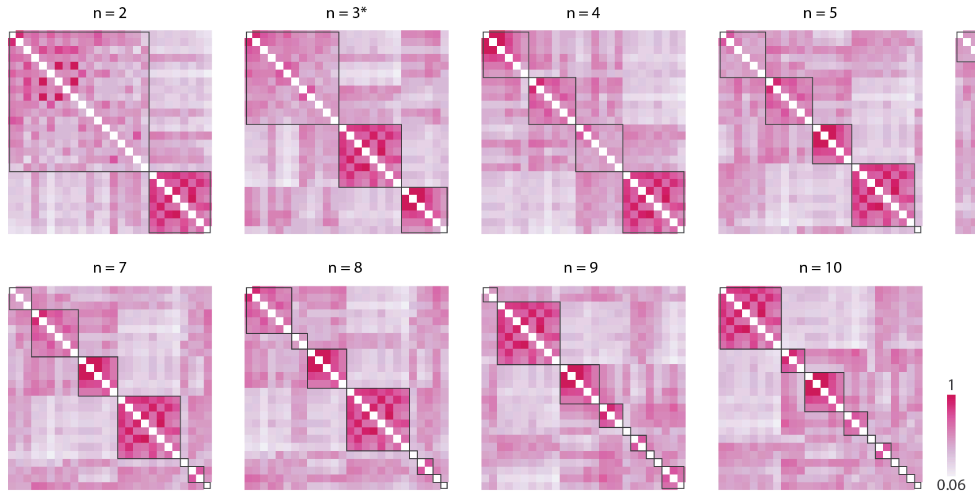

> “Long-lifetime PSD95 synapse density was correlated with each of 6,471 time-series features. A feature x feature correlation matrix (Spearman’s r) for the 26 significantly correlation time-series features was subjected to agglomerative clustering, where the number of clusters was defined from n = 2 to n = 10. We show each clustering solution here. The clustering solution shown in the Supplement of the manuscript is n = 3 (indicated by an asterisk).”

We have revised the Methods text accordingly (“Methods” section, “Time-series feature extraction” subsection, Paragraph #3):

> “For the other two synapse types, we calculate a feature x feature similarity matrix for significantly correlated features (in the case of SAP102, we also threshold these features to those where r>0.5, to reduce the set from 211 features to 59). This similarity matrix was clustered nine times using agglomerative clustering with the number of clusters defined as 2 through 10. Clustering solutions were visually inspected and one solution is shown in Fig. S6a (long-lifetime PSD95) and Fig. S5a (SAP102). This clustering solution was selected based on (1) minimizing the number of clusters with very few (<5) features, (2) maximizing the correlation within clusters, and (3) minimizing the correlation between clusters. Finally, we triangulate toward a single representative time-series feature to highlight in the main text by considering (1) correlation coefficient magnitude, (2) number of features that measure a similar property, which can be estimated via cluster size, and (3) feature explainability.”

Furthermore, we have added the following sentence in the captions of Figures S3-S6:

> “Black boxes represent clusters identified using agglomerative clustering.”

4. S5: Can the authors comment on the SY_LocalGlobal_std_* feature that seems to have the highest correlation? How is this related to the 'extreme Events' (DN_OutlierInclude) feature?

SY_LocalGlobal_std_p1 compares the standard deviation of the first 1% of the time-series (14 time points) with the standard deviation of the full time-series (1414 time points). DN_OutlierInclude is picking up on a similar phenomenon except instead of measuring standard deviation, this function considers the presence of outlier events. In other words, DN_OutlierInclude allows us to better understand why the standard deviation in local segments of the time-series may differ from the global time-series. We therefore highlight DN_OutlierInclude over SY_LocalGlobal_std_p1, because although they are measuring similar phenomena, DN_OutlierInclude is more specific.

Figure S5 caption:

> “Figure S5. Time-series features most associated with PSD95-like colocalized synapse density | We repeat the analysis in Fig. S4 using PSD95-like colocalized synapses. We generate a feature x feature correlation matrix (Spearman r) for the 41 time-series features that are significantly correlated with PSD95-like colocalized synapse density after Bonferroni correction. The correlation between each time-series feature and PSD95-like colocalized synapse density is shown in the scatter plot on the right of the matrix. The feature with the greatest correlation is SY_LocalGlobal_std_p1 which compares the standard deviation of the first 1% of the time-series with the standard

deviation of the full time-series. DN_OutlierInclude reflects a similar phenomenon except rather than measuring standard deviation DN_OutlierInclude considers the presence and timing of outlier events. In other words, DN_OutlierInclude allows us to better understand why the standard deviation in local segments of the time-series may differ from the global time-series."

5. S6: Can the authors comment on the SC_FluctAnal… feature (with also high correlation value in S3) that seems to have the highest correlation? How is this related to the stationarity of the signal?

SC_FluctAnal_2_dfa_50_logl_ssr is a function in the family of detrended fluctuation analysis methods that considers how segments of the time-series fluctuate with respect to segment size. For time-series that show regular patterns of fluctuation (e.g. heart rate variability, Gaussian noise) the output value from this function will be low. On the other hand, for time-series that are more noisy - that is, segments fluctuate a lot depending on window size - this value will be high. Signal stationarity also measures the variability of the signal across different windows, but StatAv10 only considers one window size (10% of the time-series) whereas SC_FluctAnal tests varying window sizes.

In general, many of the time-series features that are highly correlated with one another are also describing similar properties. For this reason, the precise feature that we highlight is somewhat arbitrary (except that some features are more intuitive and easier to explain than others).

Figure S6 caption:

"Time-series features most associated with PSD95-like colocalized synapse density | We repeat the analysis in Fig. S3 using PSD95-like colocalized synapses. We generate a feature x feature correlation matrix (Spearman r) for the 41 time-series features that are significantly correlated with PSD95-like colocalized synapse density after Bonferroni correction. The correlation between each time-series feature and PSD95-like colocalized synapse density is shown in the scatter plot on the right of the matrix. The time-series feature with the highest correlation is SC_FluctAnal_2_dfa_50_logl_ssr, which considers how segments of the time-series fluctuate with respect to segment size, and whether the relationship between fluctuation and segment size scales. Signal stationarity also measures the variability of the signal across different windows, but StatAv10 only considers one window size (10% of the time-series) whereas SC_FluctAnal tests varying window sizes."

6. For results shown in Fig. 3, what are the corresponding results for the 2 types of colocalized synapses?

Apologies for neglecting to include all colocalized synapse type results in the Supplement. Since the two clusters of PSD95/SAP102-colocalized synapse types are distributed very similarly to long-lifetime PSD95-exclusive and SAP102-exclusive synapses, the findings are highly consistent. We have now included a new Supplementary Figure S8 which replicates Figure 3 using colocalized synapse types.

Supplementary Figure S8:

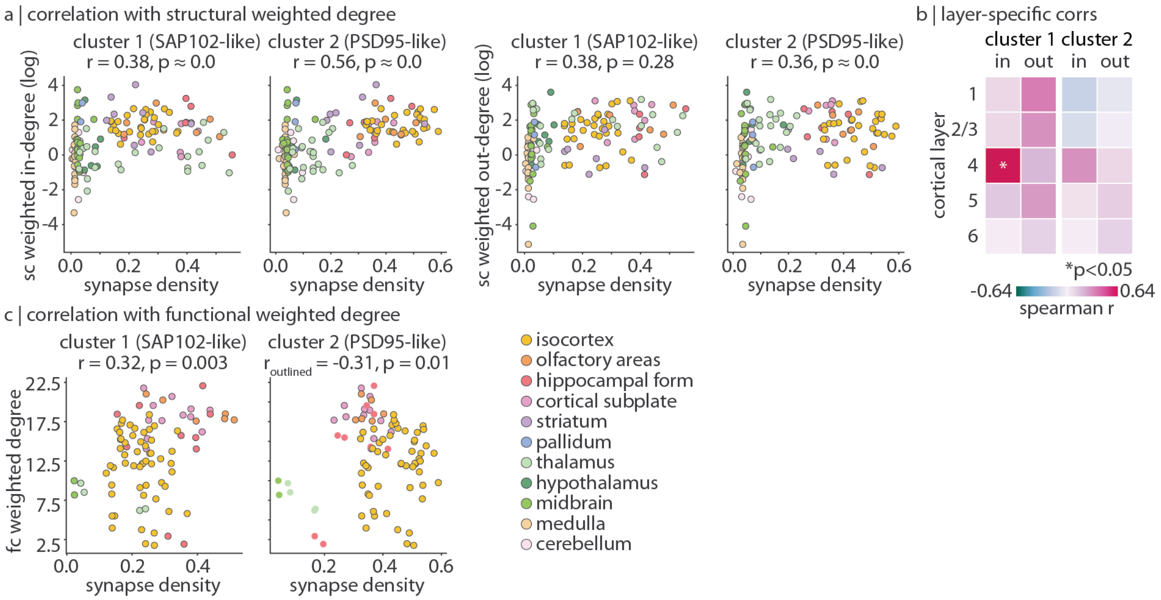

"Figure S8. Colocalized synapse types colocalize with structural and functional hubs | (a) Correlations between cluster 1 colocalized (SAP-102 like) synapses or cluster 2 colocalized (long-lifetime PSD95-like) synapse density and weighted in- or out-degree of structural connectivity (sum of all afferent or efferent connections to or from a brain region, respectively). Weighted degree (y-axis) is log-transformed for visualization. Each point is a brain region, and regions are coloured according to their ontological structure. (b) Synapse density within each of 5 layers in the isocortex were separately correlated with weighted in- and out-degree. Asterisks represent p<0.05. (c) Correlations between cluster 1 (SAP102-like) or cluster 2 (PSD95-like) colocalized synapse density and weighted degree of functional connectivity (sum of functional connectivity between one region and all other regions). Each point is a brain region, and regions are coloured according to their ontological structure. In the left-most scatter plot, only isocortical, olfactory, and cortical subplate regions are outlined and included in the correlation calculation."

7. Fig. 3f. Why did the authors pick the specific regions for the correlation calculations only for the PSD95-long case?

This selection was made post hoc. After visualizing the scatter plot between FC strength and long-lifetime PSD95 synapse density, we noticed a clear relationship between long-lifetime PSD95 synapse density and FC strength within neocortical structures (r=-0.53), but not for non-neocortical (e.g. midbrain, thalamus, some of hippocampal formation). The non-neocortical structures that do not follow the relationships between FC strength and synapse density are also the regions with nearly no long-lifetime PSD95 synapse density. As a result, we interpret this finding to indicate a relationship between synapse density and FC strength in regions where synapse density is nonzero (i.e. neocortex).

We have clarified this in the text ("Results" section, "Synapse types colocalize with structural and functional hubs" subsection, Paragraph #3):

"We next compare synapse density with functional connectivity (pairwise correlation between regional fMRI time-series). Specifically, we correlate synapse density with regional weighted degree of functional connectivity, a measure of functional integration (defined as the sum of functional connectivity strengths between one region and all others; Fig. 3f). We find that short-lifetime PSD95 synapses are not correlated ($r=-0.08$, $p=0.46$), and SAP102 synapses are weakly correlated ($r=0.31$, $p=0.01$), with functional weighted degree. On the other hand, when we visualize the correlation between long-lifetime PSD95 synapse density and functional weighted degree, we find a strong negative correlation within cortical (isocortical, olfactory, and cortical subplate) structures ($r=-0.53$, $p \sim 0$), that is, when excluding structures with near-zero synapse density (e.g. midbrain, thalamus, part of hippocampus). In other words, regions with fewer (but non-zero) long-lifetime PSD95 synapses (and more stationary signal) tend to be functional hubs."

8. How are the results for the synapse-informed model for structure-function coupling if the two colocalized types are included as independent variables?

In the main text, we consider two models: one where FC is predicted from SC alone, and one where FC is predicted from SC and one synapse type of interest. We find that only short-lifetime PSD95 synapses significantly improve SC-FC coupling during the awake compared to anaesthetized state (i.e. only short-lifetime PSD95 $p < 0.05$). In this new analysis, we build one model that predicts FC from SC, and a second model that predicts FC from SC plus colocalized synapse density plus the synapse density of interest (ie. long-lifetime PSD95, short-lifetime PSD95, or SAP102 synapses). We see the same effect for all synapse types: the addition of synapse information in the model increases SC-FC coupling significantly more in the anaesthetized versus awake state. This suggests that the effect of the individual non-colocalized synapse is minimal in comparison to the effect of adding colocalized synapses to the model, which is expected given the similar spatial distribution of colocalized synapses and long-lifetime PSD95 and SAP102 synapses.

("Results" section, "Synapse types mediate structure-function relationships" subsection, Paragraph #3):

"Specifically, we recompute structure-function coupling before and after the addition of the three synapse types using a functional connectivity matrix derived from anaesthetized mice (Fig. 4b). We then compare how the addition of synapse type changes the regional distribution of ΔR^2_{adj} for awake versus anaesthetized mice (Fig. 4c). For long-lifetime PSD95 and SAP102 synapses, structure-function coupling increases are not significantly different in anaesthetized versus awake mice (Wilcoxon signed-rank $p > 0.05$). Short-lifetime PSD95 synapses on the other hand demonstrate a significantly reduced ΔR^2_{adj} in anaesthetized mice, that is, short-lifetime PSD95 synapse density information improves structure-function coupling significantly more in awake versus anaesthetized mice (Wilcoxon signed-rank $p<0.00$; for ΔR^2_{adj} stratified by functional system, see Fig. S9). For completeness, we include colocalized synapses in the synapse-informed model and find ΔR^2_{adj} is significantly increased (Wilcoxon signed-rank $p < 0.05$) in anaesthetized versus awake mice, suggesting first that colocalized synapses dominate the change in structure-function coupling, and second that colocalized synapses contribute a similar but stronger effect as long-lifetime PSD95 and SAP102 synapses (Fig. S10)."

Supplementary Figure S10:

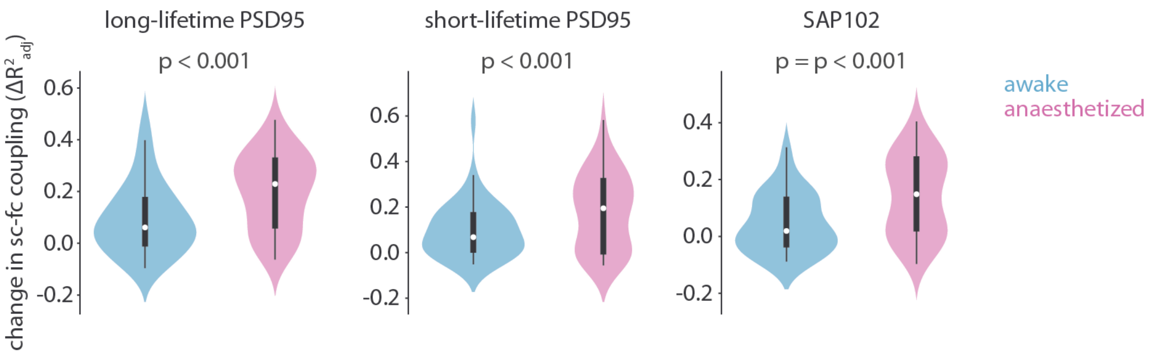

"Figure S10. Colocalized synapse type density improves structure-function coupling more in anaesthetized mice | For each brain region, we predict its functional connectivity profile from either regional communicability alone (a measure of structural connectedness) or regional communicability alongside three synaptic variables: cluster 1 colocalized synapse density, cluster 2 colocalized synapse density, and either long-lifetime PSD95, short-lifetime PSD95, or SAP102 synapse density. Structure-function coupling is defined as the fit (ΔR^2_{adj}) of the model. We compare the change in structure-function coupling (distribution of ΔR^2_{adj}) in awake versus anaesthetized mice. Statistical significance is assessed using a two-sided dependent non-parametric t-test for paired samples (Wilcoxon signed-rank test). Violin plots estimate a kernel density on the underlying data, the white point represents the median, the thick vertical line represents the quartiles of the distribution, and the thin vertical line represents the range."

9. S8: All three synapse types induce a change in structure-function coupling (though in different ways, with long-lifetime PSD95 and SAP102 showing an increase and short-lifetime PSD95 showing a decrease in anesthetized mice). Can the authors comment on these differences across the different anesthesia?

We thank the Reviewer for prompting us to explore more deeply the differences between the two anaesthetic states. In the original analysis, we find that short-lifetime PSD95 synapses significantly improve SC-FC coupling in the awake versus anaesthetized (with halothane) state. We posit that the short lifetime of these PSD95 synapses are ideal for moment-to-moment cognitive state changes that react to environmental cues, and are therefore most relevant to functional activity when the mouse is awake. We find the same significant increase in SC-FC coupling in awake mice from short-lifetime PSD95 synapses when mice are anaesthetized with a combination of medetomidine and isofluorane ("med-iso"). However, in the med-iso state, rather than observing non-significant decreases in ΔR^2_{adj} from long-lifetime PSD95 and SAP102 synapses between anaesthetized and awake states, we find *significant* decreases (though the change in ΔR^2_{adj} is minimal).

This difference may be driven by subtle differences in fMRI dynamics between the two anaesthetic states. For example, med-iso has been shown to shift the spectral components of BOLD fluctuations towards higher frequencies (Grandjean et al 2020), while halothane better preserves the spectral properties of BOLD fluctuations (Gutierrez-Barragn et al 2019).

("Results" section, "Synapse types mediate structure-function relationships", Paragraph #4):

“Finally, to ensure generalizability across different anaesthetics, we replicate these findings using a separate dataset of mice anaesthetized with a combination of medetomidine and isofluorane (N=14, Fig. S11). Short-lifetime PSD95 synapses again significantly increase structure-function coupling in awake mice; however, the decrease in structure-function coupling in awake mice for long-lifetime PSD95 synapses and SAP102 synapses are now statistically significant. This change may reflect subtle differences in fMRI dynamics between the two anaesthetic states. For example, medetomidine/isofluorane has been shown to shift the spectral components of BOLD fluctuations towards higher frequencies (Grandjean et al 2020), while halothane better preserves the spectral properties of BOLD fluctuations (Gutierrez-Barragn et al 2019).”

Grandjean, J., Canella, C., Anckaerts, C., Ayrancı, G., Bougacha, S., Bienert, T., ... & Gozzi, A. (2020). Common functional networks in the mouse brain revealed by multi-centre resting-state fMRI analysis. *Neuroimage*, *205*, 116278.

Gutierrez-Barragan, D., Basson, M. A., Panzeri, S., & Gozzi, A. (2019). Infraslow state fluctuations govern spontaneous fMRI network dynamics. *Current Biology*, *29*(14), 2295-2306.

10. "PSD95 and SAP102 synapse density are derived from a single male mouse, raising the concern that these synapse density maps are specific to the individual We therefore use gene expression data from in situ hybridization experiments in the Allen Mouse Brain Atlas to test whether synapses measured in a single mouse are coexpressed with synaptic genes measured across multiple mice". Although the results shown in Fig. 5 are very interesting and provide functional insights into the different synapse types, this analysis does not address the single male mouse concern of the dataset, as it does not validate the results shown in Fig. 1. The dataset of Bulovaite et al., 2022, which includes multiple animals and results correlate with the clustering result of the single-animal data could maybe partially address this concern? I suggest the authors to rephrase this statement to highlight the importance of the results shown in Fig. 5.

We agree with the Reviewer and apologize for misplacing the insights that are derived from Figure 5. We have rephrased the sentence as follows:

(“Results” section, “Sensitivity and robustness analysis” subsection, Paragraph #1-2):

“In this final subsection, we conduct six analyses to gauge the sensitivity and robustness of the current findings: (1) we confirm synapse density data is coexpressed with synapse-related gene expression data from a larger population of mice, (2) we confirm our findings are not driven by fMRI SNR, (3) we ensure fMRI signal amplitude and time-series features are not driven by motion, (4) we confirm our findings remain consistent when global signal regression is applied to the fMRI signal, (5) we assess the replicability of regional time-series phenotypes and spatial time-series feature value distributions, and (6) we confirm that these results are specific to synapse types as opposed to spatial variation in cell types.

First, PSD95 and SAP102 synapse density are derived from a single male mouse, raising the concern that these synapse density maps are specific to the individual and are not generalizable to a broader population. Due to technological limitations, high spatial resolution synapse density data is not available for a larger population of animals. While this hinders our ability to validate synapse density maps, we instead apply gene

expression data from in situ hybridization experiments in the Allen Mouse Brain Atlas to test whether synapses measured in a single mouse are coexpressed with synaptic genes measured across multiple mice (Lein et al 2007)."

11. For the results shown in Fig. 5, what are the corresponding results for the 2 types of colocalized synapses? Do they follow the long-lifetime PSD95 and SAP102 profiles?

We have included a new Supplementary Figure S13 which shows the gene ontology associations for both PSD95/SAP102 colocalized synapse types. Colocalized synapses also demonstrate maximal correlation with synapse associated genes, similar to PSD95 and SAP102 profiles.

Supplementary Figure S13:

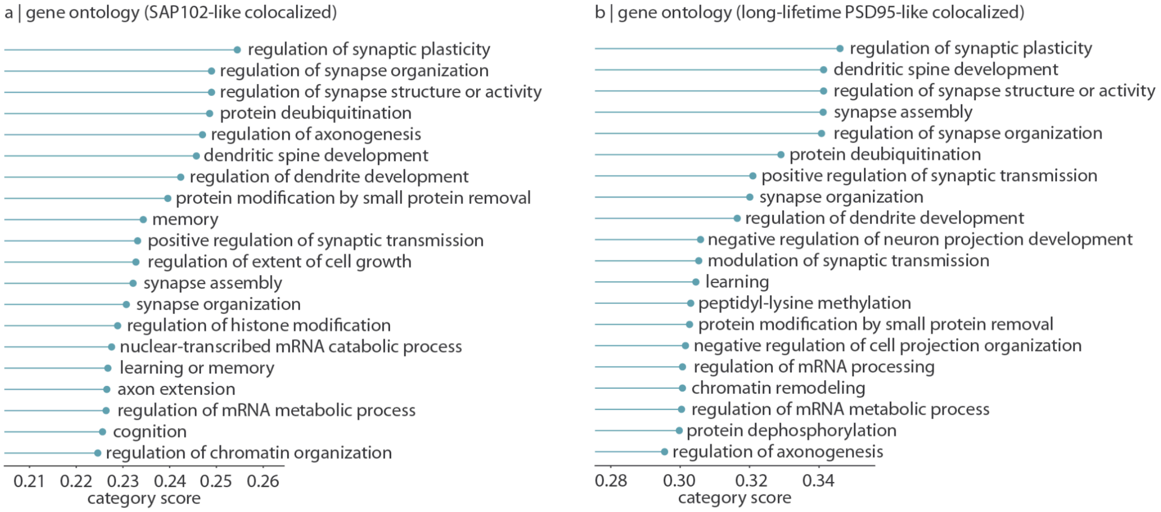

"Figure S13. Transcriptomic profiling of colocalized synapse types | Gene expression data was acquired from the Allen Mouse Brain Atlas and correlated with colocalized synapse type density. (a--b) For each of 1,616 biological process categories associated with at least 100 genes, we calculate the median absolute correlation (Spearman's r) between cluster 1 colocalized (SAP102-like) (a) or cluster 2 colocalized (long-lifetime PSD95-like) (b) synapse density and all genes in the category ("category score", x-axis). The top 20 category scores for each synapse type are shown."

12. Why did the authors use data only from female mice from the study of Bulovaite et al., 2022?

In the original study, data from only female mice were successfully acquired for simultaneous PSD95 and PSD95+SAP102 colocalized synapses. We used the full dataset and did not exclude any mice from the analysis.

13. Do the authors have insights, based on their results, regarding the role of the colocalized long-lifetime PSD95- and SAP102-like synapse types that could be included in the Discussion section? Also, the use of only male mice for the fMRI data could be commented as limitation of both the analyzed datasets and produced data.

We have added a new paragraph in the Discussion focused on interpreting the role of colocalized synapses ("Discussion" section, Paragraph #8):

> "Synapse diversity, arising from the many possible combinations of postsynaptic proteins, creates a large repertoire for synaptically-coded physiological responses (Frank et al 2016, Zhu et al 2018). It is therefore puzzling that, rather than synergistically creating a new synapse type with a unique physiological and haemodynamic profile, PSD95-SAP102 colocalized synapses appear to be redundant copies of either PSD95-exclusive or SAP102-exclusive synapses. Previous work has shown that the molecular composition of PSD95 synapses are reprogrammed in mice that lack SAP102 synapses, perhaps in a compensatory action (Frank et al 2016, Zhu et al 2018, Grant 2019). Colocalized synapses may therefore provide the molecular "option" to reprogram a synapse from PSD95 to SAP102, or vice versa. On the one hand, synaptic reprogramming may only be necessary in the extreme case of genetic mutation that renders a synapse type damaged (Grant 2019). However, it is also possible that colocalized synapses are able to dynamically adjust their physiological response profile, according to external input. Overall, studying synaptome reprogramming in colocalized synapses will help create a better understanding of the purpose and function of colocalized synapses."

Furthermore, we have edited multiple locations in the text to more directly address the fact that synapse density data are derived from a single mouse.

("Introduction", Paragraph #4):

> "Using whole-brain quantifications of PSD95- and SAP102-expressing synapses in a single mouse brain, alongside awake resting-state functional magnetic resonance imaging (fMRI) recordings in a separate population of 10 mice, we compare the spatial distribution of synapse types to >6,000 features of regional fMRI time-series."

("Results" section, Paragraph #1):

> "Approximately one billion individual synaptic puncta were imaged in a ~~whole~~ single mouse brain, using fluorescent markers for two synaptic proteins: PSD95 and SAP102 (Zhu et al 2018)."

("Results" section, "Sensitivity and robustness analysis" subsection, Paragraph #2):

> "First, PSD95 and SAP102 synapse density are derived from a single male mouse, raising the concern that these synapse density maps are specific to the individual and are not generalizable to a broader population. Due to technological limitations, high spatial resolution synapse density data is not available for a larger population of animals. While this hinders our ability to gauge the generalizability of synapse density maps, we instead apply gene expression data from in situ hybridization experiments in the Allen Mouse Brain Atlas to test whether synapses measured in a single mouse are coexpressed with synaptic genes measured across multiple mice (Lein et al 2007)."

("Discussion" section, Paragraph #10):

> “Second, although whole-brain synapse mapping technology is both recent and state-of-the-art, current whole-brain synapse density maps are limited to a single male mouse. This is an important limitation that hinders our ability to assess whether the current findings can be generalized to a broader population of animals or across sexes. As synapse mapping technology becomes more high throughput, it will become possible to gauge the generalizability of synapse density maps, as well as study how synaptic architecture influences individual-specific structure, function, and behaviour.”

Minor comments:
14. Consider including line numbers in the manuscript to assist the review process.

Done.

15. Page 1: refs [1,4,5] do not follow order of appearance (refs 2,3 are not referenced before).

Thanks for catching this. This is due to Figure 1 being placed above the Introduction text in the .tex file. This will be corrected during the editorial proofing stage.

16. Fig. 1b. Indicate in the figure legend what the square boxes represent in the plot, as well as the clustering method (as done in the Supplementary Figures). Also, highlight that this and all similar figures are symmetric (or consider showing half of the matrix).

Done.

Figure 1 caption:

> “Heatmap (symmetric): pairwise spatial correlation (Spearman's r) of synapse density. Black squares represent clusters identified using the Louvain community detection algorithm.”

Reviewer #3:

This manuscript by Hansen et al. presents an ambitious study that aims to bridge microscale synapse diversity with macroscale brain dynamics using fMRI in the mouse. The integration of large-scale synaptome mapping, functional imaging, and connectivity analysis represents a bold and creative attempt to link levels of brain organization that are rarely studied together. The topic is timely, and the dataset is rich. That said, I have several comments and concerns that I believe should be addressed to strengthen the manuscript.

1. It is not fully clear whether this work should be primarily considered a methodological resource paper (emphasizing the integration of large datasets and computational approaches) or with novel discoveries to bridge cross-scale brain anatomy and function. At present, the study feels somewhat in-between. A clearer articulation of scope—resource versus discovery-driven—would help readers and clarify the intended contribution.

Thank you for bringing this to our attention. While our work does apply large datasets and computational approaches, the primary aim is to better understand the relationship between the brain's local biology and global dynamics. We have edited the manuscript throughout to better clarify the discovery-driven nature of our work.

Abstract:

> "Here we ~~derive~~ map the spatial distribution of multiple synapse types to >6,000 time-series features from fMRI recordings in awake mice to ~~construct a~~ understand the comprehensive dynamical phenotype of ~~multiple~~ synapse types."

"Introduction" section, Paragraph #1:

> "~~Characterizing~~ Incorporating the brain's synaptic heterogeneity ~~different synapse phenotypes, or synapse "types", throughout the brain~~ is necessary for understanding how regional synaptic architecture ultimately shapes regional brain dynamics and connectivity."

"Discussion" section, Paragraph #1-2:

> "In the present study, we find that different properties of neural activity can be traced back to the expression of specific synapse types. As a result, the synaptic composition of a brain region not only shapes its macroscale dynamics but also its embedding in whole-brain structural and functional networks. Synapse lifetime emerges as an important property of the synapse, whereby long-lifetime synapses tend to be related to brain connectivity while short-lifetime synapses are involved in functional processing during wakefulness. Altogether, this work bridges the microscale and the macroscale in describing synaptic influence on whole-brain dynamics and connectivity.
>
> The present report is part of a broader effort to understand how different synapses shape brain function. Although synapses are traditionally thought to belong to one of three groups (excitatory, inhibitory, modulatory), mass spectrometry and immunoblotting experiments have revealed an enormous diversity of synaptic proteins and synaptic functions (Zhu et al 2018, Orourke et al 2012, Collins et al 2006). Here we focus on two

types of excitatory synapses: those that express PSD95 and those that express SAP102. These two proteins are differentially expressed throughout the brain and have different effects on the synapse (Zhu et al 2018). While the structure and function of individual synapse types have been described (Cuthbert et al 2007, Gomperts et al 1996, Dosemeci et al 2007), their macroscale impact is less well understood (Zhu et al 2018, Nithianantharajah et al 2013). The fundamental question that we address is: is the synaptic make-up of a brain region related to its functional dynamics?"

"Discussion" section, Paragraph #11:

"In summary, ~~we present synapse distribution as a novel molecular feature with direct influence on regional dynamics~~ we demonstrate that local synapse morphology is robustly associated with properties of regional fMRI dynamics. As a result, the synaptic composition of a brain region will affect its participation in whole-brain structural and functional networks, as well as in different behavioural states. Altogether, this work illustrates the fundamental role of synapses in shaping whole-brain organization."

2. The reported associations between long-lifetime PSD95 synapses and stationarity, or between SAP102 synapses and high-amplitude events, are intriguing. However, the mechanistic link between synapse architecture and rs-fMRI signal fluctuation remains underdeveloped. For example, it is unclear how specific synaptic distributions translate into region-specific neuronal activity patterns and ultimately into hemodynamic variability. The unresolved challenge of disentangling vascular versus neuronal contributions to rs-fMRI signals further complicates their interpretation. A more explicit discussion of these limitations and how they might be addressed in future work would be valuable.

We have modified the Discussion to include a deeper exploration of the mechanistic link between single-synapse morphology and regional dynamics, as well as future experiments that would support and extend the present findings.

("Discussion" section, Paragraph #4–5):

"One overlooked yet compelling molecular feature that likely modulates regional activity is the synapse (Wang et al 2020). Here we compare regional dynamical phenotypes to the underlying expression of different synapses and find that each synapse type is associated with specific features of brain activity. Pharmacological experiments at the level of the single synapse have established that synapse types are associated with different excitatory postsynaptic potentials (EPSPs) (Migaud et al 1998, Cuthbert et al 2007). It therefore follows that patterns in macroscale dynamics emerge as a sum of synapse-specific EPSPs. Indeed, we find that PSD95 synapses are enriched in regions with non-stationary dynamics and SAP102 synapses are enriched in regions whose dynamics are marked by high-amplitude events. This suggests that SAP102 synapses are tuned to co-incident input stimuli that result in a burst of activity, whereas PSD95 synapses are responsive to a wide range of inputs, resulting in more variable excitatory postsynaptic potentials, firing rate, and macroscale neural dynamics. Our finding is consistent with reports from single-synapse pharmacological experiments that show that PSD95 synapses are involved in synapse strengthening regardless of the input stimulus frequency (Migaud et al 1998), whereas SAP102 synapses are frequency-specific in their modulation of plasticity (Cuthbert et al 2007). Altogether, this supports the notion that the

overlapping distributions of heterogeneous synapse types, each associated with specific neuronal response properties, may combine to encode diverse types of inter-regional signaling and generate unique spatiotemporal dynamics (Zhu et al 2018).

The present report relates local synaptic morphology to statistical properties of regional haemodynamics, but does not experimentally address intermediate steps. This work would therefore benefit from multi-scale experiments that seek to understand how combinations of synapses generate unique population dynamics. For example, extending single-synapse pharmacological experiments to neuronal populations with a controlled synaptic composition would aid in establishing how individual EPSPs are summed over a neuronal population to generate specific characteristics of population dynamics. Likewise, replicating this study with alternative, more direct measurements of neural activity (e.g. calcium imaging in mice, or magneto/electro-encephalography (M/EEG) in humans) would establish whether the reported time-series feature associations can be generalized to other imaging and recording technologies each with their own biological interpretation. Finally, this work is complemented by a rich literature on neurovascular coupling that seeks to understand the relationship between electrical population dynamics and haemodynamics (Theriault et al 2023, Masamoto et al 2012), including reports that neurovascular coupling is cell- (and possibly synapse-) specific (Uhlirova et al 2016). We hope that our findings will initiate deeper exploration into the mechanistic relationship between regional synaptic composition and population dynamics."

3. The authors acknowledge that synapse density, tract-tracing, and fMRI data were derived from different mouse populations. From a rodent fMRI perspective, a key priority would be to establish the reproducibility of the spatial distribution of the >6,000 extracted temporal features across animals. Such evidence would strengthen confidence in the cross-scale association-related discoveries rather than dataset-specific effects.
In summary, this is a bold and inspiring piece of work that pushes boundaries by attempting to link microscale synapse architecture with macroscale brain dynamics.

We thank the Reviewer for raising this point and agree that establishing the robustness of the spatial distributions of time-series features is important. Despite considerable progress in the field, awake rodent fMRI remains challenging and time-courses are therefore considerably noisier than in anaesthetized mice. Indeed, when we calculate the pairwise correlation of temporal feature brain maps for every pair of mice, we find an average correlation of r=0.13. We therefore opted to average temporal features across mice to maximize signal. When we compare the spatial profiles of temporal features in individual mice with that of the average spatial profile, we find a considerably increased correlation (mean rho = 0.43).

To illustrate this further, we estimate the mean reproducibility of the group-average spatial distributions of temporal features. From our sample of N=10 awake mice, and for every N = {2, 3, 4, 5}, we construct two size-N subgroups of mice. We then calculate the average spatial distribution of all temporal features across every distinct pair of N = {2, 3, 4, 5} mice. We then correlate the group-average temporal feature density across groups. (For example, if N=5, we (1) split the sample of 10 mice into every combination of 2 groups of 5 (252 combinations), (2) calculate the mean spatial profile of time-series features within each group of 5 mice, (3) correlate the group-mean spatial profiles for every pair of groups, and (4) calculate the mean correlation (i.e. mean reproducibility) across all subsample pairs and all features.) We plot the average correlation (i.e. mean reproducibility) of group-mean temporal feature distributions as a function of the size of the group. Next, we fit these four data points to a logarithmic curve (R^2 = 0.9998) and predict

that, for our sample of 10 mice, the mean reproducibility of group-mean temporal feature distributions is 0.4165.

While a mean time-series feature reproducibility of 0.4165 is encouraging in the context of awake rodent fMRI, we nonetheless address the limitations of time-series feature reproducibility and small sample size in the manuscript.

Finally, we also assess the reproducibility of time-series features by comparing regional time-series phenotypes across mice (see also our response to Reviewer #1 Comment #7). For every brain region (rows) and mouse (columns), we correlate the full regional time-series profile of the mouse with the average regional time-series profile. We show the correlation coefficients in the heatmaps below. All individual time-series phenotypes are positively correlated with the mean time-series phenotype, with the most variable brain region being correlated on average at r=0.33 and the least variable brain region being correlated on average at r=0.68.

("Results" section, "Sensitivity and robustness analysis" subsection, Paragraph #7):

> "Fifth, we assess the reproducibility of time-series features by comparing individual-mouse phenotypes to group-average phenotypes. For every brain region and mouse, we correlate the full regional time-series phenotype profile of the mouse with the average regional time-series phenotype profile (6,471 x 1 vector of time-series feature values) (Figure S17). All individual time-series phenotypes are positively correlated with the mean time-series phenotype (range of mean correlation: [0.33, 0.68]). Next, for every time-series feature and mouse, we correlate the spatial distribution of time-series feature with the average time-series feature. The empirical mean correlation between individual and group time-series spatial distributions is 0.43 (see Methods for more details)."

("Discussion" section, Paragraph #10):

> "Similarly, time-series feature calculation was conducted on fMRI data from a small sample of 10 mice, and individual reproducibility of the spatial distribution of time-series features is poor (mean r=0.13). We therefore average time-series features across mice to maximize signal and reproducibility (predicted mean r=0.42; see *Methods* for details)."

("Methods" section, "Time-series feature extraction" subsection, Paragraph #1–2):

> "For every mouse and every brain region, the corresponding time-series was subjected to an automated massive time-series feature extraction pipeline using the Highly Comparative Time-Series Analysis toolbox (*hctsa*) (Fulcher 2013, Fulcher 2017). This toolbox calculates >7,000 statistical features (e.g. mean, variance, stationarity, entropy) on each time-series and normalizes the features across brain regions to the unit interval using a scaled robust sigmoid normalization (parameter normFunction = 'mixedSigmoid'). After discarding features with zero variance across regions, we retained the subset of features present across all mice, resulting in a 88 region x 6471 feature x 10 mice matrix of time-series feature values.
>
> To assess the reproducibility of the spatial distribution of each time-series feature, we calculate pairwise spatial correlations for every time-series feature across every pair of mice. We find that time-series reproducibility is low (mean Spearman's r=0.13). We

therefore average time-series feature values across mice to maximize signal and reproducibility. Indeed, when we correlate the spatial distributions of each single-mouse time-series feature with the group-averaged spatial distribution, we find increased reproducibility (mean Spearman's r=0.43). We take this one step further and assess the reproducibility of group-mean spatial distributions of time-series features as a function of sample size. More specifically, from our sample of N=10 mice, we calculate the average spatial distribution of all temporal features across every distinct combination of N = {2, 3, 4, 5} mice. For every pair of groups, we correlate their group-mean time-series feature spatial distribution, and finally calculate the average across all pairwise correlations (i.e. mean reproducibility). We find that mean reproducibility increases with sample size logarithmically (R^2 = 0.9998) and predict that the mean reproducibility when N=10 is 0.42 (see Figure S20). We therefore only analyze group-mean time-series features, resulting in a 88 region x 6,471 feature matrix of time-series feature values (shown in Fig. 2c)."

Figure S20:

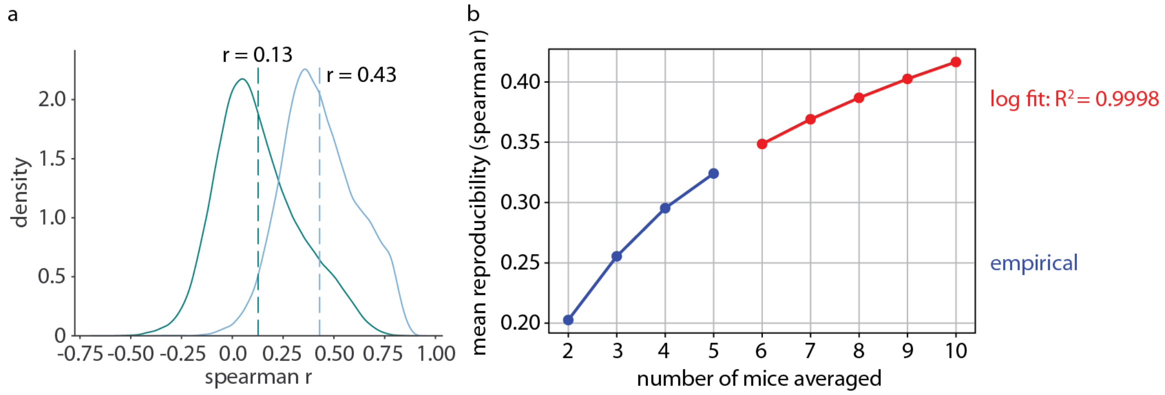

"Figure S20. Inter-individual variability of time-series feature distributions across brain regions | (a) Green distribution: pairwise Spearman correlation of time-series feature spatial distribution for every pair of mice (10 mice total = 45 pairs) and all time-series features (6,471 features). Mean correlation (green dashed line) is 0.13. Blue distribution: Spearman correlation between group-average time-series feature spatial distribution and individual time-series feature spatial distribution, for all time-series features (6,471 features) and mice (10 mice). Mean correlation (blue dashed line) is 0.43. (b) For N in {2, 3, 4, 5}, we select all possible combination of 2 size-N subgroups of mice, average time-series feature spatial profiles within subgroups, and correlate the two subgroups. The average correlation across time-series features and subgroups ("reproducibility") is shown on the y-axis. Next, we fit a logarithmic function on the empirical mean reproducibility (model fit R^2 = 0.9998) and predict the mean reproducibility for up to 10 mice."

Figure S17:

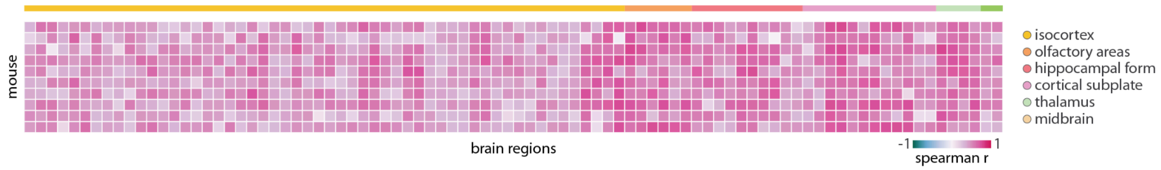

“Figure S17. Each brain region is associated with a time-series phenotype (6,471 x 1 vector of time-series values). This heatmap represents the Spearman correlation between each region’s group-averaged time-series phenotype with the time-series phenotypes from individual mice. Brain regions are ordered according to ontological structure, as indicated by the vertical bar and the legend.”
