# Supplementary material for: Synapse types are spatially associated with regional hemodynamics in the mouse brain
Source: PLoS Biol. 2026 Feb 12;24(2):e3003637. doi: 10.1371/journal.pbio.3003637 (PMC12919930; doi:10.1371/journal.pbio.3003637)
Supplement: Attachment — Submitted filename: Hansen_Response_to_Reviwer.pdf [file pbio.3003637.s023.pdf]

Dear Dr. Hart and Reviewer,

Thank you for the conditional acceptance of our revised submission. We feel the revision process was fair and considerably improved the manuscript. Here we respond to the remaining Reviewer comment, and make minor changes to the manuscript. Reviewer comments are in **bold font** and our responses are in regular font.

Reviewer #3:

The authors' responses are thoughtful, but a potential conceptual gap remains in the interpretation of resting-state fMRI signals. In particular, the manuscript gives limited consideration to hemodynamic and vascular contributions to rs-fMRI, despite aiming to link synaptic architecture to fMRI dynamics. Several emphasized features of fMRI signal fluctuations in this manuscript are known to be sensitive to vascular and hemodynamic variability, yet the interpretation remains largely neuronal. I am not requesting additional analyses, but a clearer acknowledgment of these factors would help balance the narrative and better define the limits of the proposed synapse-fMRI link.

We apologize for the lack of exposition on the limitations of hemodynamic measurements as a proxy for neuronal signal. We have expanded on this point in a paragraph in the Discussion as well as in the limitations paragraph.

("Discussion" section, Paragraph #5):

> "The present report relates local synaptic morphology to statistical properties of regional haemodynamics, but does not experimentally address intermediate steps. Given the reported findings are derived from fMRI, which is influenced not only by neuronal activity but also by vasculature (Attwell & Iadecola 2002, Kim & Ogawa 2012), elucidating the neuronal component of the synapse-dynamics relationship remains an important goal. This work would therefore benefit from multi-scale experiments that seek to understand how combinations of synapses generate unique neuronal population dynamics. For example, extending single-synapse pharmacological experiments to neuronal populations with a controlled synaptic composition would aid in establishing how individual EPSPs are summed over a neuronal population to generate population dynamics. Likewise, replicating this study with alternative, more direct measurements of neural activity (e.g. calcium imaging in mice, or magneto/electro-encephalography (M/EEG) in humans) would establish whether the reported time-series feature associations can be generalized to other imaging and recording technologies each with their own biological interpretation. This work would also be complemented by a deeper understanding of the relationship between synapse density and variations in regional blood flow, or more generally on neurovascular coupling, for the purposes of establishing neuronal specificity of synapse-dynamics relationships. For example, a replication analysis using time-varying measurements of cerebral blood flow would reveal whether synapse density is uniquely correlated with hemodynamic signal or if synapse density is similarly associated with variability in regional blood flow. While the relationship between neuronal activity and vascular variability is not the focus of this manuscript, we note that interpreting synapse-hemodynamic relationships is incomplete without considering neurovascular coupling (Theriault et al 2023, Masamoto et al 2012, Uhlirova et al 2016). We hope that

our findings will initiate deeper exploration into the relationship between regional synaptic composition and neuronal population dynamics."

("Discussion" section, Paragraph #10):

"Fourth, the present report uses fMRI dynamics as a proxy for neural dynamics, despite known vascular contributions to the fMRI signal (Attwell & Iadecola 2002, Kim & Ogawa 2012). The reported findings are therefore not reflective of purely neuronal activity."
